# Certificated Dispensers and the Insurance Bill

**Published:** 1911-11-11

**Authors:** 


### CERTIFICATED DISPENSERS AND THE INSURANCE BILL.

Sir,—I have read with pleasure the letter of "An Apothecary's Assistant," but I regret that he, in his reference to the Association of Certificated Dispensers, Apothecaries' Hall, London, accuses the Association and the Honorary Secretary of inaction in the present crisis. As the active work of the Association takes place in London, much is done which members living in distant parts are necessarily unacquainted with. As a member I know we need an organ which we may look upon as representative, and which may sound the trumpet note when necessary. Your correspondent is aware of the powerful forces opposed to us. Victory is generally with those who have the longest guns and the most money. Our friends the enemy have both. In these circumstances every certificated dispenser should be a member of the official Association, and should loyally support the executive. All the work done by the officials is honorary. We fight for justice, we ask for justice; believing that England is England still, we hope for justice. "Collega."

